# Adult neurogenesis in the ventral hippocampus decreased among animal models of neurodevelopmental disorders

**DOI:** 10.3389/fncir.2024.1504191

**Published:** 2024-12-23

**Authors:** Lihao Sun, Nobuhiko Ohashi, Takuma Mori, Yuka Mizuno, Weichen Zang, Qi Guo, Emi Kouyama-Suzuki, Yoshinori Shirai, Katsuhiko Tabuchi

**Affiliations:** ^1^Department of Molecular and Cellular Physiology, Shinshu University School of Medicine, Matsumoto, Japan; ^2^Department of Neuroinnovation, Institute for Biomedical Sciences, Interdisciplinary Cluster for Cutting Edge Research, Shinshu University, Matsumoto, Japan

**Keywords:** adult hippocampal neurogenesis, prenatal nicotine exposure, valproic acid, IQSEC2, neuroligin 3, autism spectrum disorder

## Abstract

**Introduction:**

Autism spectrum disorder (ASD) is a neurodevelopmental condition characterized by deficits in social interaction and communication, along with restricted and repetitive behaviors. Both genetic and environmental factors contribute to ASD, with prenatal exposure to valproic acid (VPA) and nicotine being linked to increased risk. Impaired adult hippocampal neurogenesis, particularly in the ventral region, is thought to play a role in the social deficits observed in ASD.

**Methods:**

In this study, we investigated social behavior and adult hippocampal neurogenesis in C57BL/6J mice prenatally exposed to VPA or nicotine, as well as in genetically modified ASD models, including IQSEC2 knockout (KO) and NLGN3-R451C knock-in (KI) mice. Sociability and social novelty preference were evaluated using a three-chamber social interaction test. Adult hippocampal neurogenesis was assessed by BrdU and DCX immunofluorescence to identify newborn and immature neurons.

**Results:**

VPA-exposed mice displayed significant deficits in social interaction, while nicotine-exposed mice exhibited mild impairment in social novelty preference. Both IQSEC2 KO and NLGN3-R451C KI mice demonstrated reduced adult neurogenesis, particularly in the ventral hippocampus, a region associated with social behavior and emotion. Across all ASD mouse models, a significant reduction in BrdU+/NeuN+ cells in the ventral hippocampus was observed, while dorsal hippocampal neurogenesis remained relatively unaffected. Similar reductions in DCX-positive cells were identified in VPA, nicotine, and NLGN3-R451C KI mice, indicating impaired proliferation or differentiation of neuronal progenitors.

**Discussion:**

These findings suggest that impaired adult neurogenesis in the ventral hippocampus is a common hallmark across ASD mouse models and may underlie social behavior deficits. This study provides insight into region-specific neurogenic alterations linked to ASD pathophysiology and highlights potential targets for therapeutic interventions.

## 1 Introduction

Autism spectrum disorder (ASD) is a group of developmental disorders defined by the presence of persistent deficits in social communication and social interaction across different situations (Hirota and King, 2023). The prevalence has been estimated from 0.4 to 4.0% and is four to five times higher in males than females (Bougeard et al., 2021; Salari et al., 2022). High heritability and the recurrence of ASD within families strongly suggest that genetic factors play a significant role in its etiology. ASD is categorized into syndromic and non-syndromic forms. Syndromic ASD is associated with comorbidities, such as intellectual disability, epilepsy, or language impairment, and is often linked to genetic abnormalities or monogenic alterations (Genovese and Butler, 2020). For instance, Fragile X syndrome, Rett syndrome, and Phelan-McDermid syndrome, are caused by the monogenic mutations in FMR1, MECP2, and SHANK genes, respectively (Masini et al., 2020; Bicker et al., 2021). Furthermore, environmental factors such as prenatal drug and chemical exposure in the mother have also been implicated in the development of ASD (Mandy and Lai, 2016; Sarieva and Mayer, 2021; Barrett et al., 2024).

Genetic studies have identified over 1,200 gene variants associated with ASD in the Simons Foundation Autism Research Initiative database (Banerjee-Basu and Packer, 2010). Among these, IQSEC2 (Intelligence quotient motif and SEC7 domain containing 2) and Neuroligin (NLGN) 3, located on the X chromosome, are prominent candidates. IQSEC2 has been identified in four families with missense mutations at Xp11.22, associated with severe intellectual disability (Shoubridge et al., 2010). Approximately 25–80 percent of patients with IQSEC2 mutations also exhibit autistic features (Levy et al., 2019; Leoncini et al., 2023). IQSEC2 belongs to a family of guanine nucleotide exchangers localized at the postsynaptic density of excitatory synapses. The activation of ADP ribosylation factor 6 by the SEC7 domain contributes to neuronal transmission through postsynaptic α-amino-3-hydroxy-5-methyl-4-isoxazolepropionic acid receptor and facilitates the development and maturation of the dendritic spines. IQSEC2 knockout (KO) and transgenic mice with the A350V mutation exhibit ASD-like social deficits (Hinze et al., 2017; Levy et al., 2019; Mehta et al., 2021). The *NLGN3* gene is implicated in a non-syndromic monogenic form of ASD (Quartier et al., 2019). The R451C substitution in NLGN3 was first identified in two siblings in a Swedish family diagnosed with autism and Asperger syndrome (Jamain et al., 2003). NLGN3, a member of transmembrane cell adhesion proteins, is localized at the post-synapse and interacts with presynaptic proteins, such as neurexins. It is expressed at both excitatory and inhibitory synapses, playing a crucial role in synaptic function and neural connectivity. The R451C mutation, a variant extensively studied, is estimated to occur in less than 3% of non-syndromic ASD cases (Quartier et al., 2019). Mice carrying the R451C missense variant in NLGN3 (NLGN3-R451C) show ASD-related behavior alterations with repetitive behavior and reduced sociability (Tabuchi et al., 2007; Gioia et al., 2023).

Environmental factors, such as prenatal exposure to valproic acid and nicotine, also contribute to the risk of ASD through epigenetic mechanisms (Masini et al., 2020). Valproic acid (VPA) is a well-established drug to treat epilepsy and psychiatric disorders (Perucca and Mula, 2013). It influences gene expressions in neurodevelopment by relieving histone deacetylase (HDAC)-dependent transcriptional repression (Larner et al., 2021; Alavi et al., 2024). Early prenatal exposure to VPA can lead to cognitive impairments and increase the risk of ASD in offspring (Bromley et al., 2008; Christensen et al., 2013; Wiggs et al., 2020). Similarly, maternal smoking during pregnancy has been linked to higher risk of ASD (Jung et al., 2017; Caramaschi et al., 2018; von Ehrenstein et al., 2021). Nicotine from tobacco readily crosses the placenta and reaches the fetus (Hellström-Lindahl and Nordberg, 2002; Lin et al., 2022). Among subunit combinations of 12 nicotine acetylcholine receptors (nAChRs), α2β4 nAChRs are most prominently expressed, and α7 nAChRs are widely distributed in the developing mammalian brain (Gotti et al., 2007; Zoli et al., 2015). Nicotine activates these receptors, disrupting cholinergic signaling and inducing epigenetic changes, such as increased histone acetylation and decreased histone methylation (Muenstermann and Clemens, 2024). Rodent models exposed to VPA or nicotine administration during pregnancy have demonstrated ASD-like behaviors, including deficits in social interaction (Schneider and Przewłocki, 2005; Alkam et al., 2013; Kataoka et al., 2013; Mabunga et al., 2015; Wang et al., 2015; Larner et al., 2021; Sivasangari and Rajan, 2022; Zhou et al., 2024).

The diagnosis of ASD is challenging due to the heterogeneity of clinical presentations, varying levels of severity, and the presence of comorbid disorders, as well as the reliance on the examiner’s subjectivity and competence (Hausman-Kedem et al., 2018; Wiggins et al., 2020; Gesi et al., 2021; Fusar-Poli et al., 2022). To address these challenges, extensive studies have aimed to identify potential biomarkers. Recent research suggests higher serum concentrations of γ-Aminobutyric acid and lower oxytocin levels in the ASD group compared to the healthy control group (Lin et al., 2023). Additionally, trace elements such as iron, copper, and zinc, which are essential for brain development and synaptic function, have been implicated in individuals with ASD (Zhang et al., 2021). Magnetic resonance imaging studies have provided structural and functional abnormalities that point to the diagnosis of ASD (Rafiee et al., 2022). The volumetric analysis demonstrated cortical thickening in the frontal lobe during the early stages of ASD, followed by thinning in the temporal cortices, including the hippocampus. Functional imaging highlights hypoconnectivity in task-specific brain regions, along with compensatory hyperconnectivity to bypass these under-connected areas (Guo et al., 2024). These suggest that the underlying neuropathology of ASD may involve abnormal brain network organization and disrupted neuronal connectivity. The function and the structure of neural circuits underlying the pathophysiology of ASD have also been investigated using ASD model animals (Sato et al., 2023). Recently, more evidence has been accumulated to indicate an association between ASD phenotypes and dysfunction of neural circuits in emotion-related brain regions, such as the medial prefrontal cortex, the amygdala, and the hippocampus. In addition, the role of adult hippocampal neurogenesis has also emerged as a critical area of investigation (Liu et al., 2023; Barón-Mendoza et al., 2024; Chen et al., 2024; Long et al., 2024).

Adult neurogenesis is a process of brain development that involves the continuous generation of new neurons throughout the lifespan (von Bohlen und Halbach, 2011; Kempermann et al., 2015). This process is restricted in two regions: the subventricular zone and the subgranular zone (SGZ) of the dentate gyrus (DG) in the hippocampus (Gage, 2000; Ming and Song, 2011). In the SGZ, radial glia-like stem cells give rise to intermediate neural progenitor cells (NPCs) with high proliferative capacity and then produce neuroblasts capable of mitosis. These newly generated neurons survive, differentiate, and integrate into existing brain networks, contributing to cognitive functions (Mongiat and Schinder, 2011; Aimone et al., 2014). To clarify the stages of neurogenesis, immunohistological techniques using specific antibodies have been employed. Bromodeoxyuridine (BrdU) is commonly used to label proliferating cells, while doublecortin (DCX), a microtubule-associated protein, is expressed from the neuroblast stage to postmitotic maturation. The hippocampus is divided into two distinct regions: the ventral and dorsal parts. The ventral hippocampus is particularly associated with ASD due to its strong connections with subcortical structures, such as the rostral hypothalamus and amygdala, related to behaviors such as social interaction and emotions (Liu et al., 2023). However, a region-specific analysis of adult hippocampal neurogenesis across ASD mouse models has not yet been conducted.

In this study, we examined the social behavior of C57BL/6J mice prenatally exposed to VPA and studied patterns of adult neurogenesis in the dorsal and ventral hippocampus. We then extended this approach to mice prenatally exposed to nicotine (prenatally nicotine exposed, PNE). We also studied the neurogenesis in each region of the hippocampus in genetically modified ASD mouse models, including IQSEC2 KO mice and NLGN3-R451C knock-in (KI) mice. VPA mice exhibited severe deficits in social interaction, while PNE mice showed mild disturbances in sociability. All these ASD model mice demonstrated a reduction in neurogenesis within the ventral hippocampus. Our results indicate that decreased adult neurogenesis in the ventral hippocampus is a common phenotype across ASD mouse models.

## 2 Materials and methods

### 2.1 Animals

All procedures of animal experiments were reviewed by the Committee for Animal Experiments and were finally approved by the president of Shinshu University. Pregnant female C57BL/6J mice were obtained from Japan SLC. IQSEC2 KO mice were generated by CRISPR/Cas9 as previously described (Mehta et al., 2021) and maintained under a hybrid background of C57BL6/J and 129+Ter/Sv strain because of a high mortality under pure C57BL6/J. NLGN3-R451C mutant mice were generated and the behavior characteristics were analyzed previously (Tabuchi et al., 2007; Cao et al., 2022). All mice were free with a libitum food and water and kept on 12 h light/12 h dark cycle at a temperature of 20 ± 1°C.

### 2.2 Drug treatments

**Figure 1:**
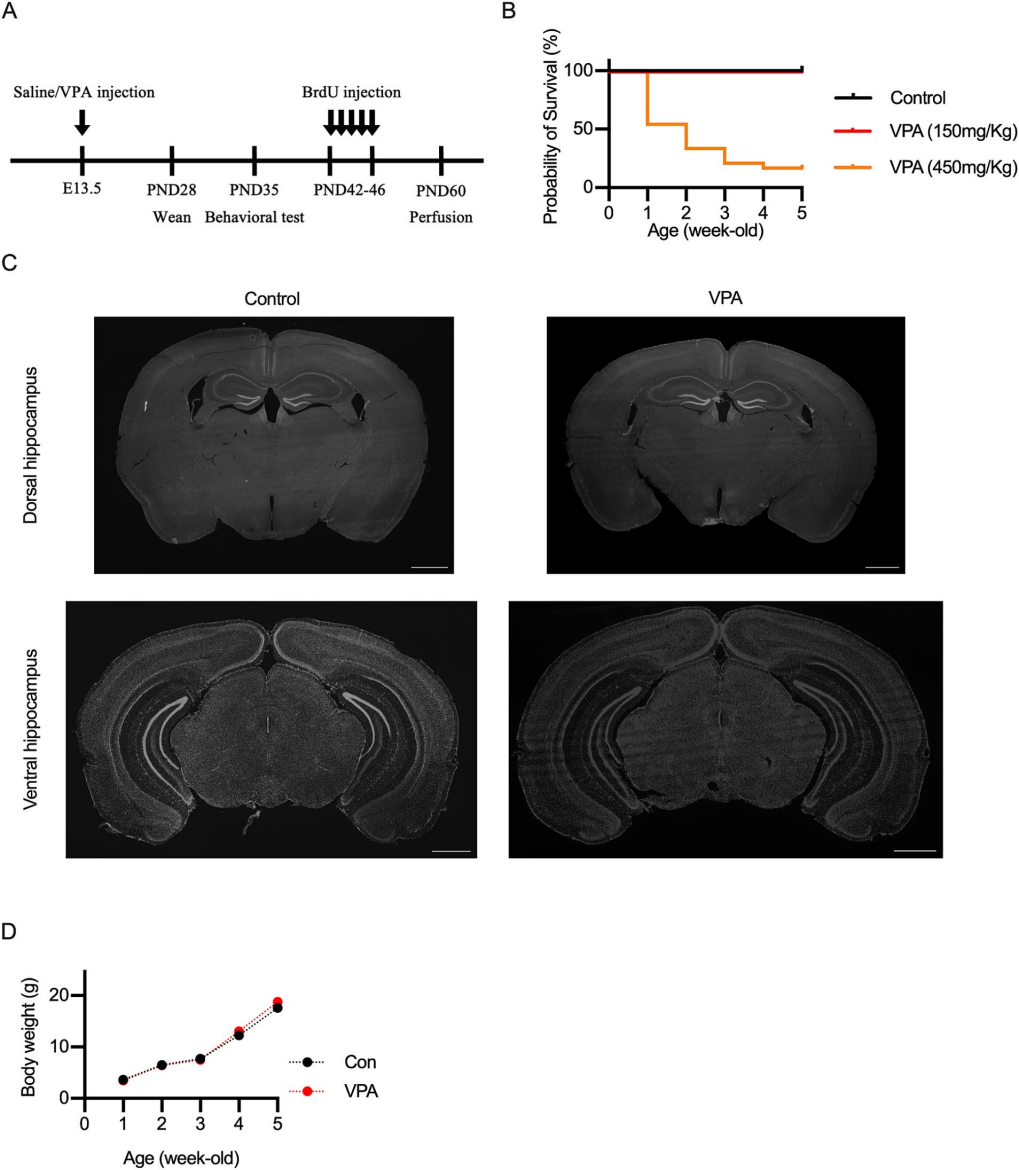
Experimental design and characterization of VPA mouse model. **(A)** Timeline of the experimental procedures, illustrating the administration of valproic acid (VPA) or saline at embryonic day (E) 13.5, followed by behavioral testing, BrdU administration, and immunohistological analysis at specific postnatal days. **(B)** Survival rate of VPA-exposed mice compared to controls. Prenatal exposure to 450 mg/kg VPA significantly decreased survival, whereas 150 mg/kg VPA showed similar survival rates to controls. **(C)** Representative DAPI-stained coronal sections of dorsal and ventral hippocampus, showing no significant morphological differences between control and VPA-exposed mice. **(D)** Body weights of VPA-exposed and control mice measured at several time points indicate no significant differences across groups. Scale bars = 1 mm. Results are presented as mean ± SEM.

To generate ASD mouse models, offspring prenatally exposed to valproic acid and nicotine were prepared. For the VPA-exposed mice model (VPA mice), we administered various dosages of VPA (150 mg/kg, 300 mg/kg, 450 mg/kg, and 600 mg/kg) intraperitoneally on embryonic day 13.5, according to previous studies (Larner et al., 2021). However, pups occasionally died shortly after birth at dosages of 300, 450, and 600 mg/kg. Consequently, a single injection of VPA (15 mg/mL dissolved in saline) at a dose of 150 mg/kg was selected for the subsequent experiments. The control group received the same dose of saline injection on the same day. Male and female pups were weighed at the postnatal day (PND) 7, 14, 21, 28, 35. Thirty-one VPA mice and 25 control mice, derived from 8 and 9 pregnant dams, respectively, were used to calculate the growth curve (Figure 1). For the nicotine-exposed mice model (PNE mice), prenatal exposure to nicotine or saline was carried out as previously described (Zhou et al., 2024). Pregnant mice received water containing nicotine (200 μg/mL) and 2% sucrose or only 2% sucrose alone from embryonic day (E) 14 until delivery. All pups were weaned on PND28, and only male mice were individually housed in separate cages until the day of the experiment.

### 2.3 Three-chamber social interaction

The behavioral test was conducted as described previously (Badawi et al., 2021; Hou et al., 2021; Zheng et al., 2024). The apparatus consists of a rectangular transparent Plexiglas box (40 cm × 60 cm × 25 cm), divided by two walls with small openings (5 cm × 3 cm), allowing access to each chamber. For VPA mice, inverted empty small black wire cups with clear glass cylinders were placed in the back-left corner of the left chamber and the front-right corner of the right chamber, while for PNE mice, the cups were positioned at the center of both chambers. The experiments were recorded with a GoPro Black 10 Black (resolution: 1,920 × 1,080 pixels, frame rate: 30 frames per second). Five-week-old males born from pregnant female mice with or without VPA were used as subjects. Non-treated four-week-old C57BL/6J male mice that had no previous contact with the subject mouse were used as strangers. All behavioral analyses were performed between 10:00 a.m. and 6:00 p.m. The subject mice were placed in the middle chamber and acclimated for 10 min of free exploration. Subsequently, the mouse was returned to the middle chamber by closing the removable doors. In the sociability test, a stranger mouse (S1) in an inverted cup was placed in the left chamber, while an empty inverted cup (E) was positioned in the right chamber. After a 10-min recording, the animals were removed from the box, and the apparatus was cleaned with 70% ethanol. In the social novelty test, S1 was placed in the same position, while a second stranger (S2) was placed in the previously empty cup, and the subject mouse was again introduced into the middle chamber. The interaction was recorded for 10 min. The exploratory behavior was defined as the subject mouse’s nose was within 2 cm of the cup. The time spent in the chamber and around the cup was manually measured from the recorded videos. Additionally, to generate the heatmaps, we utilized DeepLabCut, a markerless tracking tool, to obtain the coordinates of the mice, as previously described (Zhou et al., 2024). Fourteen VPA male mice (from eight mothers) and 13 control male mice (from nine mothers) were used for the tests, as well as nine PNE male mice (from four mothers) and nine control male mice (from three mothers). Given that ASD is more prevalent in males than in females (Bougeard et al., 2021; Salari et al., 2022), and that sex differences can significantly influence behavioral phenotypes in both human and animal models (Lai et al., 2015; Shepard et al., 2017), we exclusively used male mice in this study.

### 2.4 BrdU injection and histological analysis

Bromodeoxyuridine (BrdU) was administered according to the schedule designed in a previous study (Zhou et al., 2024). Male mice were intraperitoneally injected with BrdU at a dose of 150 mg/kg from P42 to P46 three times a day for five consecutive days. Two weeks after the last injection, the animals were used for histological analysis. All 67 brains were used for immunohistochemical quantification.

Histological analysis was followed by previous procedures (Mori and Morimoto, 2014; Mori et al., 2019; Pang et al., 2022). Mice were anesthetized with a cocktail containing medetomidine hydrochloride (Domitor, 0.3 mg/kg), midazolam (Dormicum, 4.0 mg/kg), and butorphanol tartrate (Vetorphale, 5.0 mg/kg). Mice were perfused transcardially with ice-cold PBS, followed by ice-cold 4% paraformaldehyde (PFA) in PBS. The brain was decapitated and postfixed in 4% PFA overnight. Each brain was soaked in 30% sucrose in PBS at 4°C until it sank. The brains were sectioned at 40 μm thickness using a freezing microtome. After rinsing with PBS, sections were incubated in a 1 M hydrochloride solution for 30 min at 45°C and washed with PBS for 15 min. Non-specific immunoglobulin binding was blocked by incubating the sections in a blocking buffer (PBS containing 2% donkey serum albumin and 0.3% Triton) for 60 min at room temperature. Brain sections were incubated in the blocking buffer containing the primary antibodies, rat anti-BrdU (ab6326, Abcam, 1:200 dilution), mouse anti-NeuN (MAB377, Roche, 1:1,000 dilution), and rabbit anti-DCX (ab18723, Abcam, 1:5,000 dilution) for 24–48 h at 4°C. The sections were washed three times for 5 min each at room temperature and incubated with the secondary antibodies, Alexa-488-conjugated donkey anti-mouse IgG (Thermo production, 1:400 dilution in PBS with 0.3% Triton X-100), Alexa-594-conjugated donkey anti-rat IgG (Thermo production, 1:400 dilution in PBS with 0.3% Triton X-100), and Alexa-594-conjugated goat anti-rabbit IgG (ab150080, Abcam, 1:1,000). After rinsing, brain sections were incubated with DAPI in the dark, and coverslipped.

Fluorescence images were taken with a fluorescent microscope (BZ-X800, Keyence) or a confocal microscope (SP8, Leica) equipped with a ×10 and ×20 objective lens. Digital zoom up to 3× was used to check the overlapping signals. To estimate the number of BrdU/NeuN double-positive newborn neurons in the hippocampus, we divided the hippocampus into two compartments according to a mouse brain atlas: the ventral hippocampus (Bregma −2.80 to −4.04 mm) and the dorsal hippocampus (Bregma −0.94 to −2.80 mm). The border we used here has been used in some other laboratories (Mohammad et al., 2018; Bauman et al., 2019; Ávila-Gámiz et al., 2023; Zhou et al., 2024), even though there is a variation of the border of the dorsal and ventral hippocampus used in different laboratories using electrophysiological and anatomical techniques (Botterill et al., 2021; Chockanathan and Padmanabhan, 2021).

We then quantified newborn neurons separately within each compartment. For each mouse, we analyzed six to eight sections, calculating the total number of BrdU+/NeuN+ cells in the dorsal and ventral DG separately. The total cell number was calculated by averaging the cell count per slice and multiplying it by the number of slices. The total number of newborn neurons in the whole DG was calculated by summing the counts from the dorsal and ventral segments. For the density of DCX positive cells, we quantified DCX+ newborn neurons and measured the length of the DG in both dorsal and ventral segments, as previously described (Zhou et al., 2024). For each mouse, we analyzed four to nine sections, calculating the total number of DCX+ cells in the dorsal and ventral DG separately. We then divided the total cell count by the corresponding total length of each area. The value thus obtained (cell number/mm) should reflect the density of DCX-positive immature neurons in the dorsal, ventral and whole DG separately.

### 2.5 Statistical analysis

All data in graphs were presented as means ± SEM. Student’s t-test was used for two-group comparisons. Most of the statistical analysis between manipulated and unmanipulated animals was performed by unpaired t-test. When we compared the values within a single animal group (in three chamber test), we used paired t-test. Statistical significance is indicated by asterisks (*p < 0.05, **p < 0.01, ***p < 0.001). Statistical analysis was performed using Graphpad Prism 8.

## 3 Results

### 3.1 Prenatal valproic acid exposure induces a social interaction deficit

To generate a VPA mouse model of ASD, a single injection of VPA (150 mg/kg, 300 mg/kg, 450 mg/kg, or 600 mg/kg) was administered to pregnant females at E13.5 (Figure 1A), as previously described (Larner et al., 2021). Mice administered with 300 mg/kg or 600 mg/kg died shortly after birth. Mice treated with 450 mg/kg survived for some time post-birth, but their survival rate was lower compared to control mice or those administered 150 mg/kg (Figure 1B). Therefore, we selected a dose of 150 mg/kg VPA for further experiments. Prenatal exposure to VPA could induce congenital malformations and decreased body weight in a dose-dependent manner (Rodier et al., 1996; Dufour-Rainfray et al., 2010; Kataoka et al., 2013; Sivasangari and Rajan, 2022). However, no gross abnormalities in morphology were observed (Figure 1C), and growth curves were consistent across the groups (Figure 1D).

**Figure 2:**
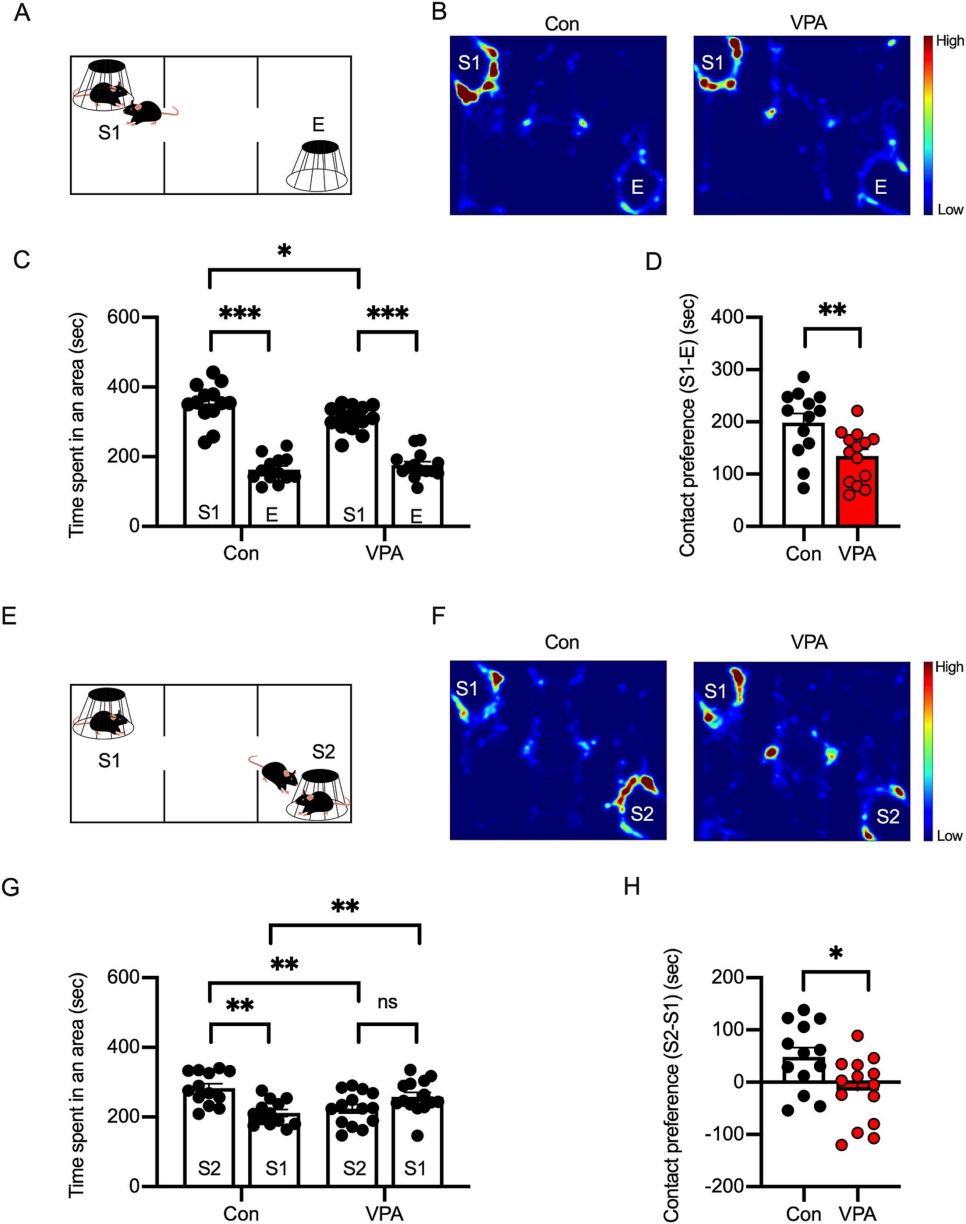
Social interaction deficits are observed in VPA-exposed mice. **(A)** Diagram of the three-chamber social preference test. S1 represents a stranger mouse, and E represents an empty cage. **(B)** Representative heatmap images depicting exploratory behavior in control and VPA-exposed mice during the social preference test. **(C)** Time spent in each area during the social preference phase indicates reduced interaction with S1 in VPA-exposed mice compared to controls. **(D)** Social preference index (S1–E) showing significantly diminished social preference in VPA mice. **(E–H)** Social novelty preference phase: diagram of the test **(E)**, heatmap images **(F)**, and quantification **(G,H)** showing reduced interaction with a novel stranger (S2) in VPA-exposed mice, suggesting impaired social novelty recognition. Results are presented as mean ± SEM, *p < 0.05, **p < 0.01, ***p < 0.001, unpaired student’s *t*-test was applied to compare Con vs. VPA; paired student’s *t*-test was applied to compare S1 vs. E.

We next evaluated the effects of prenatal VPA exposure on social interaction, a core ASD behavior, using the three-chamber social interaction test (Crawley, 2004). This test uses a box divided into three compartments through which the mouse can freely move. In each of the two end compartments, a small cup-like cage is placed. One of these cages contains a novel juvenile mouse (Stranger 1 = S1), while the other cage is left empty (E). The test mouse is placed in the central compartment, and subsequently, the time spent in S1, E, and the central compartment is recorded (Figure 2A). Wild-type mice are known to contact the stranger mouse more frequently than the empty cage, which serves as an indicator of sociability (social preference). In the sociability phase, the interaction time with a stranger (S1) was higher in control mice than in VPA mice (Figures 2B–D).

In the three-chamber test, in addition to the social preference test, the social novelty preference is also assessed. After the social preference test, a new stranger mouse (S2) is placed in the cage that was previously empty (Figure 2E). At this point, the previous stranger mouse (S1) becomes a familiar mouse, as it has already been exposed to the test mouse. During this phase, the test mouse’s contact with either the familiar mouse (S1) or the stranger mouse (S2) is compared. Typically, wild-type mice interact more with S2 than with S1, which indicates social novelty preference. In the social novelty phase, while the control mice interacted more with the new stranger (S2), VPA mice exhibited a reduced preference for S2, suggesting a social deficit in VPA mice (Figures 2F–H).

### 3.2 Adult hippocampal neurogenesis is reduced in the VPA mouse model

**Figure 3:**
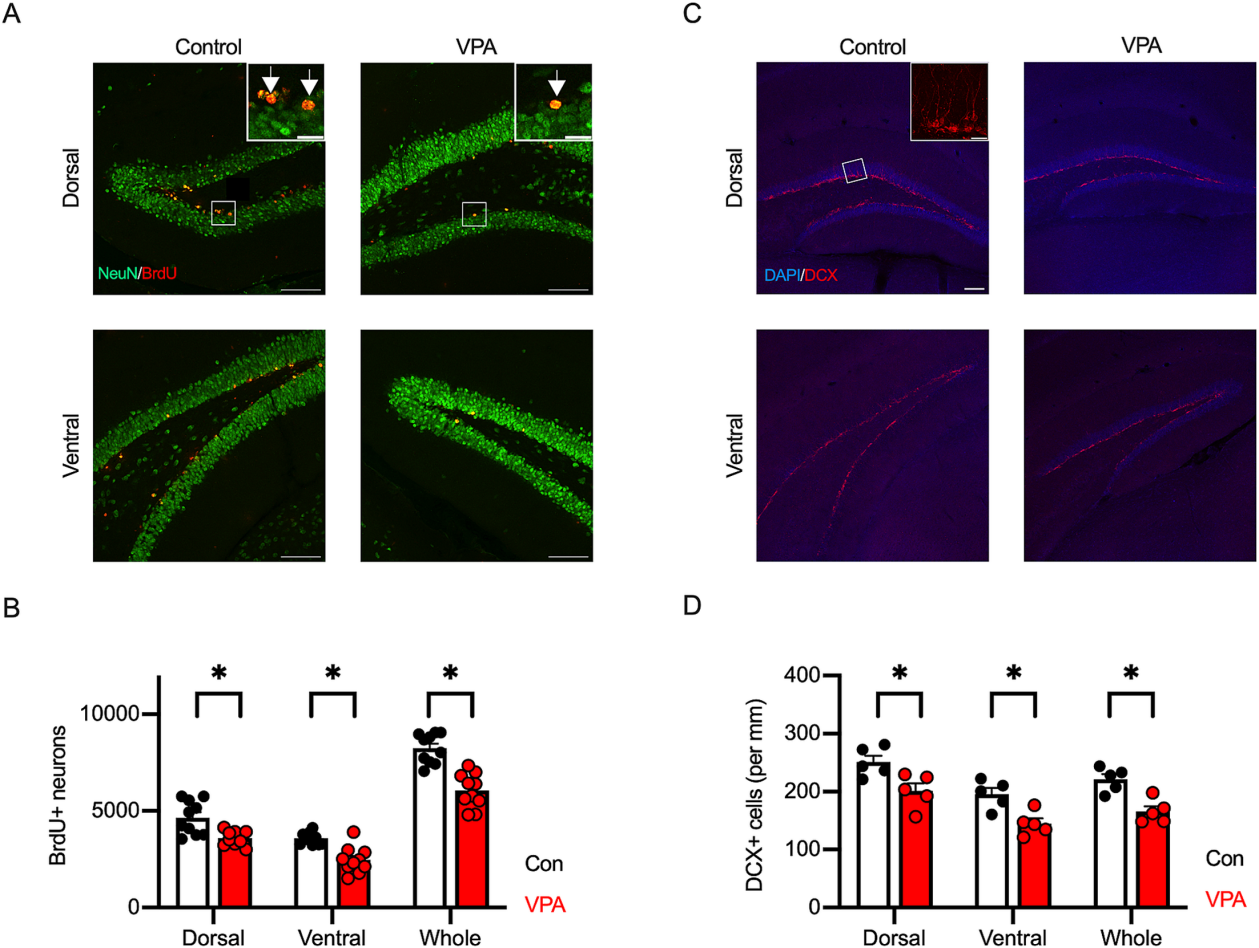
Reduction of adult hippocampal neurogenesis is observed in VPA-exposed mice. **(A)** Representative immunofluorescence images showing BrdU (red) and NeuN (green) co-labeling in dorsal and ventral hippocampus of control and VPA mice. BrdU/NeuN double-positive cells indicate newborn neurons. Inset represents zooming in of the location indicated by a square on the main image. Arrows indicate BrdU/NeuN double-positive cells. Scale bars = 100 μm or 20 μm (insets). **(B)** Quantification reveals a significant reduction in the number of BrdU+/NeuN+ cells in both dorsal and ventral hippocampus of VPA mice compared to controls. **(C)** Immunofluorescence images of doublecortin (DCX, red)-positive cells in dorsal and ventral hippocampus. The brain structure was visualized with DAPI (blue). Inset represents zooming in of the location indicated by a square on the main image. An arrow indicates DAPI/DCX double-positive cells. Scale bars = 100 μm or 20 μm (inset). **(D)** The cell density of DCX-positive immature neurons shows a significant decrease in both dorsal and ventral hippocampus of VPA mice compared to controls, indicating impaired neurogenesis. Results are presented as mean ± SEM, *p < 0.05, **p < 0.01, Student’s t-test.

Alteration of adult neurogenesis in the hippocampus has been implicated in animal models of ASD (Bicker et al., 2021; Barón-Mendoza et al., 2024). The dorsal and ventral areas of the hippocampus are thought to have different functions. The dorsal hippocampus is primarily involved in spatial cognition, while the ventral hippocampus is believed to play a role in cognitive functions related to social behaviors and emotions. This is further supported by their distinct neural projections to cortical and subcortical regions. To assess the effect of prenatal VPA exposure on adult hippocampal neurogenesis, we performed immunofluorescent labeling for BrdU and the neuron-specific marker NeuN to identify adult-born neurons in these regions. We found a significant reduction in the number of BrdU/NeuN double-positive cells in VPA mice compared to control mice across all hippocampal regions (Figures 3A,B).

Adult hippocampal neurogenesis is tightly regulated by the process of proliferation, migration, differentiation, survival, and maturation of neurons (von Bohlen und Halbach, 2011; Kempermann et al., 2015). During these stages, neuronal stem cells in the SGZ of the dentate gyrus give rise to NPCs that express the early neuron-specific marker DCX. To further clarify the stages at which impairment of adult hippocampal neurogenesis occurs, we performed fluorescence immunostaining using an antibody against DCX. As a result, DCX-positive cell density was significantly decreased throughout all hippocampal regions in VPA mice compared to control mice (Figures 3C,D). These results suggest that prenatal VPA exposure reduces adult neurogenesis in the hippocampus in mice due to a decrease in NPCs.

### 3.3 Prenatal nicotine exposure induces a social interaction deficit and impairs adult neurogenesis in the ventral hippocampus

**Figure 4:**
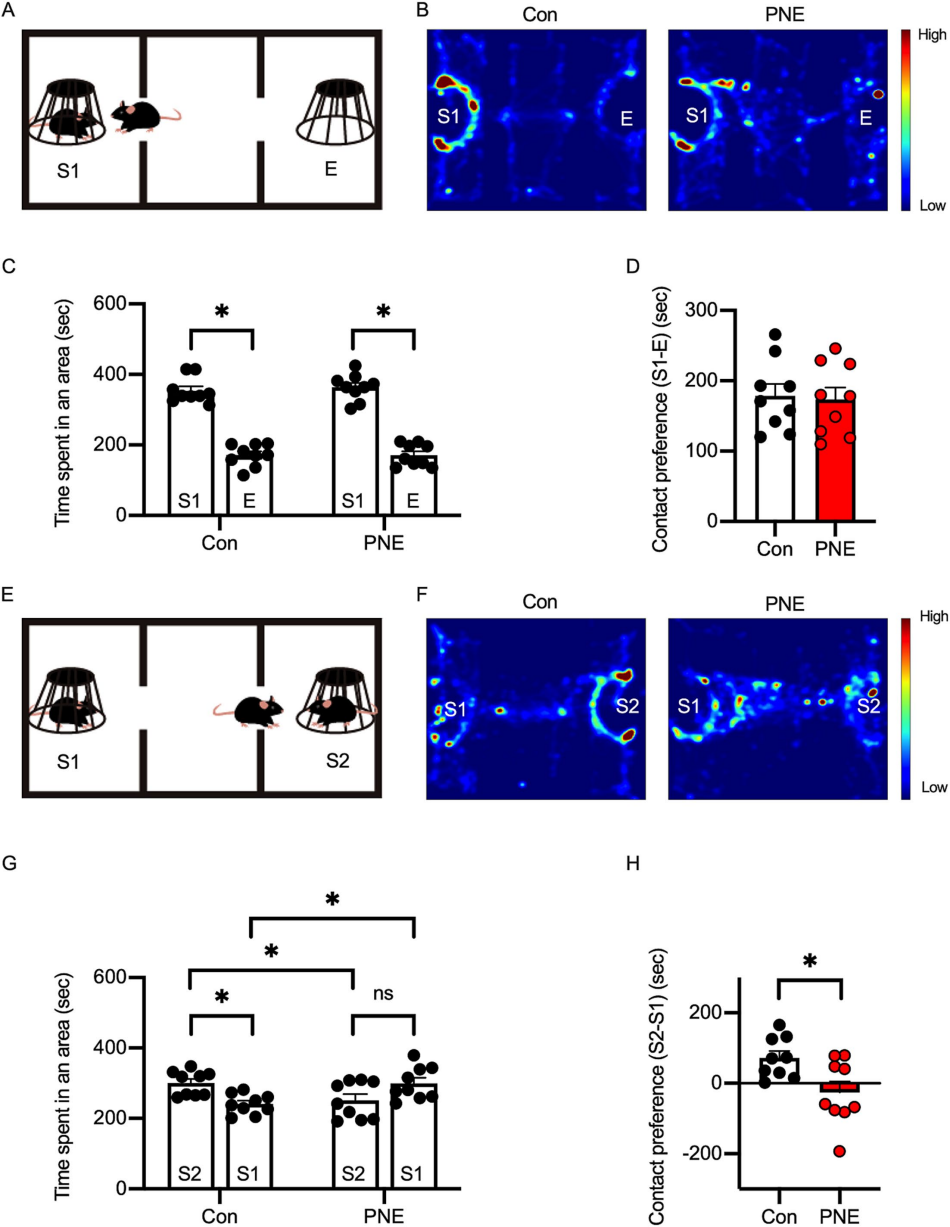
Social behavior deficits are observed in prenatal nicotine-exposed (PNE) mice. **(A)** Diagram of the social preference test setup. **(B)** Heatmap images of exploratory behavior in PNE and control mice during the social preference phase. **(C,D)** Quantification of time spent in each area **(C)** and contact preference **(D)** during the social preference phase indicates no significant difference between PNE and control mice. **(E–H)** Social novelty preference test: diagram **(E)**, heatmap images **(F)**, and quantification **(G–H)** show reduced preference for a novel stranger (S2) in PNE mice, indicating impaired social novelty recognition. Results are presented as mean ± SEM, *p < 0.05, **p < 0.01, ***p < 0.001, unpaired student’s t-test was applied to compare Con vs. VPA; paired student’s t-test was applied to compare S1 vs. E.

We previously reported that PNE mice exhibited characteristics of attention-deficit/hyperactive disorder and ASD (Zhou et al., 2024). To further investigate social deficits in PNE mice, we performed a three-chamber social interaction test (Figure 4). In the sociability phase, both control and PNE mice exhibited preference to the stranger (S1) mice than empty cage (E) (Figures 4B–D). On the other hand, in the social novelty phase, PNE group spent less interaction time with a new stranger (S2) and more interaction time with a now familiar mouse (S1) compared to control group (Figures 4F–H), suggesting that the social novelty preference was impaired in PNE mice.

**Figure 5:**
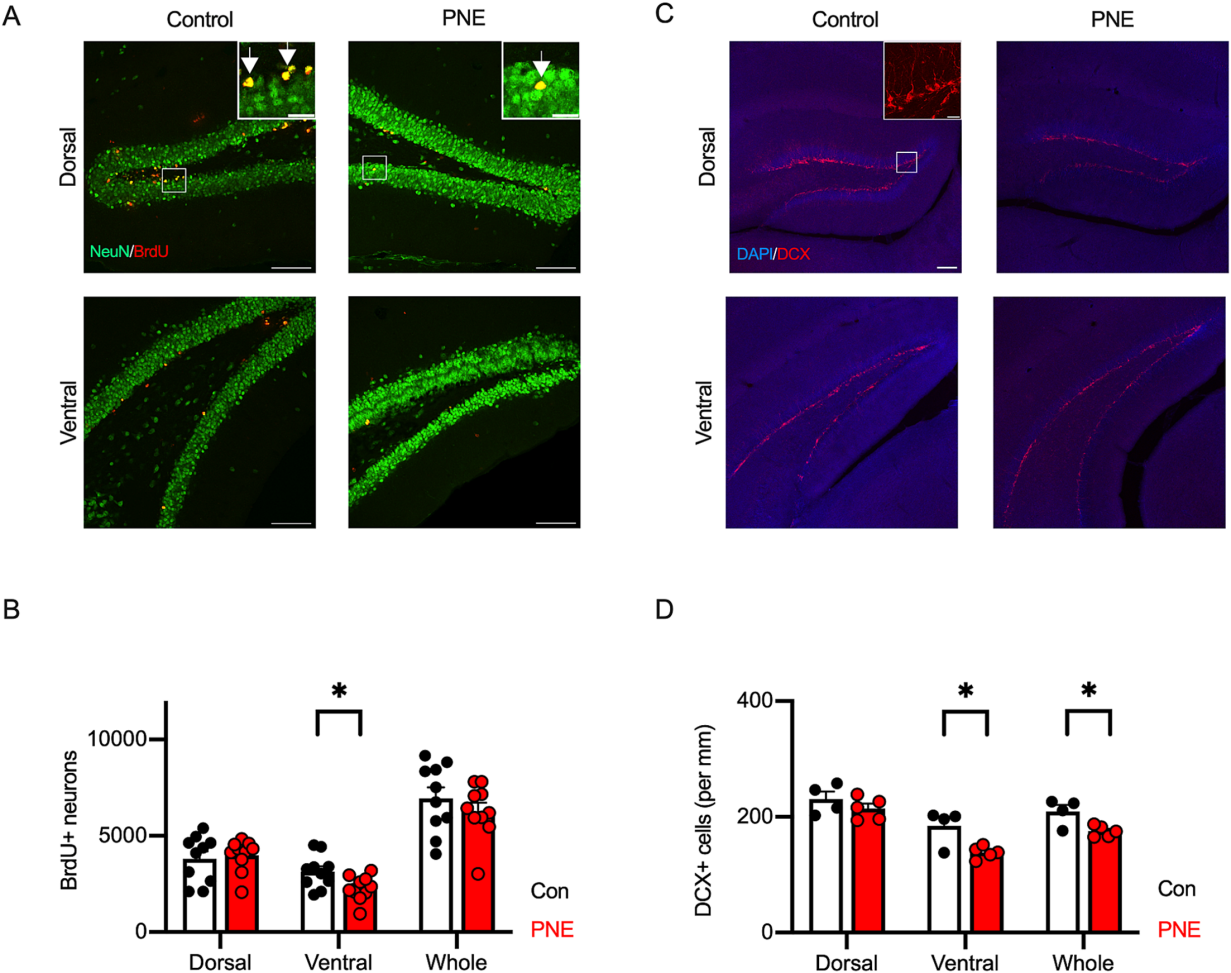
Reduction of adult hippocampal neurogenesis is selectively observed in the ventral hippocampus of PNE mice. **(A)** Representative immunofluorescence images showing BrdU (red) and NeuN (green) labeling in the dorsal and ventral hippocampus of control and PNE mice. Inset represents zooming in of the location indicated by a square on the main image. Arrows indicate BrdU/NeuN double-positive cells. Scale bars = 100 μm or 20 μm (insets). **(B)** Quantification reveals a reduction in BrdU+/NeuN+ cells in the ventral hippocampus of PNE mice compared to controls. **(C,D)** DCX staining (red) **(C)** and quantification **(D)** show reduced cell density of DCX-positive immature neurons (per mm) in the ventral hippocampus of PNE mice. In Figure 5C, the brain structure was visualized with DAPI (blue). Inset represents zooming in of the location indicated by a square on the main image. An arrow indicates DAPI/DCX double-positive cells. Scale bars = 100 μm or 20 μm (inset). Results are presented as mean ± SEM, *p < 0.05, **p < 0.01, Student’s t-test.

We next evaluated adult neurogenesis in the dorsal and ventral areas of hippocampus in PNE mice. PNE mice exhibited a decreased number of newborn neurons (BrdU+/NeuN+) in the ventral hippocampus compared to controls (Figures 5A,B). We did not detect the differences between control and PNE mice in dorsal area of hippocampus (Figures 5A,B). These results were consistent with our previous study in PNE mice (Zhou et al., 2024). Cell density of DCX+ immature neurons was reduced in the ventral, not in the dorsal, hippocampus in PNE mice (Figures 5C,D).

### 3.4 IQSEC2 KO and NLGN3-R451C KI mice show impaired adult neurogenesis in the ventral hippocampus

**Figure 6:**
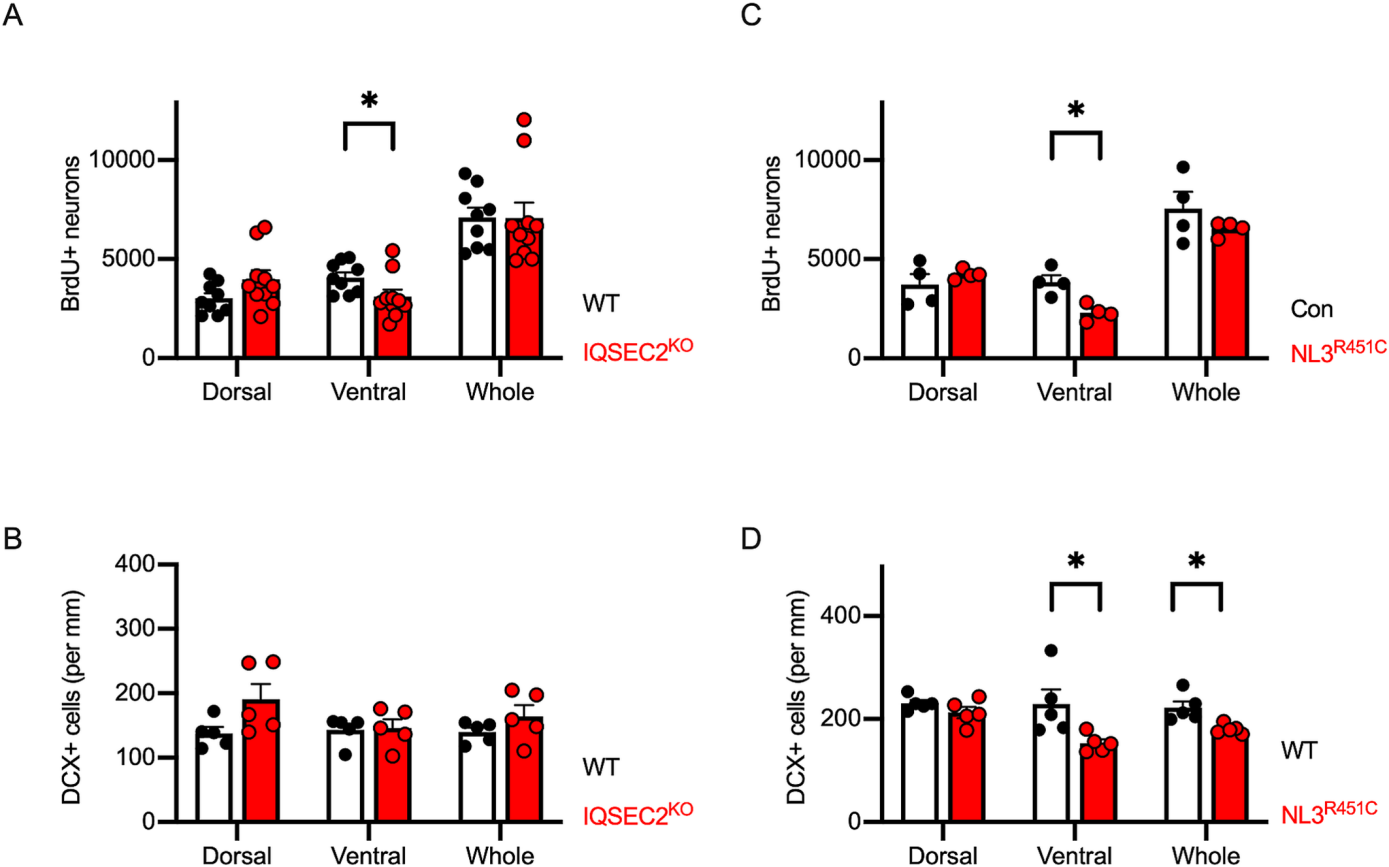
Reduction of adult hippocampal neurogenesis is selectively observed in the ventral hippocampus of IQSEC2 KO and NLGN3-R451C KI mice. **(A,C)** Quantification of BrdU+/NeuN+ double-positive cells in the hippocampus of IQSEC2-KO **(A)** and NLGN3-R451C KI **(C)** mice. Reduction in BrdU+/NeuN+ cells is observed in the ventral hippocampus of both mouse models. **(B,D)** Quantification of DCX-positive cells in the hippocampus of IQSEC2-KO **(B)** and NLGN3-R451C KI **(D)** mice. DCX-positive cell density was reduced in the ventral hippocampus of NLGN3-R451C KI mice compared to WT controls. Results are presented as mean ± SEM, *p < 0.05, **p < 0.01, Student’s t-test.

Based on these results, we hypothesized that impaired adult neurogenesis in the ventral hippocampus is a common characteristic in ASD model mice. Both IQSEC2 KO mice and NLGN3-R451C KI mice exhibited autistic behaviors in our previous studies (Tabuchi et al., 2007; Mehta et al., 2021; Cao et al., 2022). To address the hypothesis, we employed the same strategies using BrdU, NeuN, and DCX immunostaining on IQSEC2 KO and NLGN3-R451C KI mice. In IQSEC2 KO mice, the number of BrdU/NeuN double-positive cells significantly decreased in the ventral hippocampus (Figure 6A). However, the cell density of DCX positive cells was similar between the dorsal and ventral areas (Figure 6B). In NLGN3-R451C KI mice, the number of BrdU+/NeuN+ cells was significantly decreased in the ventral, but not dorsal, hippocampus (Figure 6C). The density of DCX positive cells was also selectively decreased in the ventral hippocampus in NLGN3-R451C KI mice (Figure 6D). These results suggest that the reduction of adult hippocampal neurogenesis in the ventral area may be the common phenotype in autistic model mice.

## 4 Discussion

In this study, we conducted the comprehensive and region-specific evaluation of adult hippocampal neurogenesis in the ventral and dorsal regions across various ASD mouse models exhibiting social interaction deficits. VPA mice exhibited severe ASD-like behaviors, characterized by reduced social preference and social novelty preference in the three-chamber test, and impaired adult hippocampal neurogenesis in both dorsal and ventral DG regions. Furthermore, we found that PNE mice exhibited a reduced social novelty preference, and that the adult hippocampal neurogenesis was selectively impaired in the ventral DG, consistent with our previous findings (Zhou et al., 2024). To our knowledge, no previous studies have examined adult hippocampal neurogenesis in IQSEC KO mice. In this study, we observed the region-specific impairment of adult hippocampal neurogenesis in IQSEC2 KO mice and NLGN3-R451C KI mice.

Since Rodier et al. (1996) successfully developed the VPA-exposure mouse model, VPA mice have been widely used as models for ASD Typically, 300–600 mg/kg of VPA is commonly administered to pregnant females at E12.5–13.5 (Larner et al., 2021; Alavi et al., 2024). However, teratogenic effects, such as ear and tail malformations, are observed in rodents subjected to 500 or 600 mg/kg (Dufour-Rainfray et al., 2010; Favre et al., 2013; Kataoka et al., 2013; Sivasangari and Rajan, 2022), and high mortality of dams exposed to 600 mg/kg has been reported in rats (Sabers et al., 2014), although significant mortality in offspring at 300–600 mg/kg is uncommon. In our study, we initially administrated various dosages of VPA intraperitoneally on E13.5. Unexpectedly, high mortality was observed, prompting us to use the lower dosage of 150 mg/kg for subsequent experiments. This observed mortality may be attributed to species differences, the administration route, the duration of VPA exposure, and its broad HDAC inhibitory activity (Larner et al., 2021). Additionally, the effects of VPA on neuronal development may vary depending on the timing of VPA administration during embryonic days 12.5–14.5 (Schneider and Przewłocki, 2005; Kataoka et al., 2013). By using 150 mg/kg, we excluded the fatal and morphological effects of the drug on offsprings.

A deficit in sociability is a core feature of ASD and has been studied in the VPA mouse models (Kataoka et al., 2013; Fujimura et al., 2016; Larner et al., 2021). We observed the decreased social preference and social novelty preference in three-chamber test in our lower dosage VPA exposure model mice. This indicates that the low-dosage VPA method may be more suitable to create simple ASD model with minimal neurological complications.

Adult hippocampal neurogenesis is a complex and dynamic process involving multiple stages, regulated by various intrinsic and extrinsic factors. Dysregulation of the neurogenesis in the hippocampus has been proposed as an underlying mechanism of ASD (Bicker et al., 2021; Liu et al., 2023; Barón-Mendoza et al., 2024). VPA, an HDAC inhibitor, has the potential to disrupt neurogenesis by altering transcriptional regulation. Juliandi et al. reported that prenatal exposure to VPA caused an increase in immature newborn neurons in the hippocampus at E14.5, which depleted the NPCs pool and led to impaired neurogenesis at PND91 (Juliandi et al., 2015). Kinjo et al. found that continuous intraperitoneal VPA injections during embryonic development increased newly born neurons at P30 in both the anterior and posterior DG (Kinjo et al., 2019). Similarly, Watanabe et al. observed an increase in newborn mature neurons at PND77 following prenatal VPA exposure (Watanabe et al., 2019). VPA dose-dependently reduces cell proliferation and induces cell differentiation in the NPCs through the upregulation of G1-phase cyclin-dependent kinase inhibitors, without affecting apoptosis (Hsieh et al., 2004; Juliandi et al., 2015; Fujimura et al., 2016). These findings suggest that prenatal VPA exposure may initially stimulate neurogenesis in the hippocampus during early development, but ultimately impair adult neurogenesis as result of a rebound phenomenon. In our study, we observed a decrease in the number of newborn neurons and a reduction in the cell density of immature neurons in DG. This could result from a later phase of neurogenesis disruption, where the timing of depletion may be influenced by VPA dosage.

PNE mice, induced by administering nicotine to pregnant animals, have been widely used as models for ASD. Nicotine impacts the developing hippocampus by dysregulating cholinergic function through binding α2β4 and α7 nAChRs, which are highly expressed in the hippocampus (Zeid et al., 2018). Although the effects of nicotine on adult neurogenesis remain unclear, α7 nAChRs play a critical role in the survival, maturation, and integration of adult-born neurons in the DG, as demonstrated by the decreased survival rates and abnormal dendritic structure in new neurons of α7KO mice (Campbell et al., 2010). In the current study, we observed that PNE mice showed less preference for a new stranger than the control mice, consistent with results from previous study (Alkam et al., 2013). We also confirmed in the previous study that PNE model mice exhibit autistic behaviors, such as increased anxiety and deficits in social interactions (Zhou et al., 2024). Both studies revealed impairments in adult neurogenesis in PNE mice, a key feature associated with ASD. Given these results, the PNE mouse model may be a strong candidate for ASD research. However, it is also noteworthy that long-term nicotine exposure and subsequent withdrawal may enhance adult hippocampal neurogenesis, warranting further investigation (Cohen et al., 2015).

The hippocampus is functionally segmented into two compartments: the dorsal segment is related to spatial navigation and memory, and the ventral segment is related to stress and emotion. The ventral region is particularly relevant to ASD-like characteristics due to its connections with the amygdala, hypothalamus, and medial prefrontal cortex (Guo et al., 2024). However, histological studies focusing on these regions separately are scarce. In this study, we found that the adult neurogenesis in the ventral DG was attenuated across various ASD mouse models, despite the distinct monogenic and epigenetic etiologies. Supporting our results, Gioia et al. reported that a reduction in neuroblast within the ventral hippocampus was associated with social behavior deficits in NLGN3-R451C KI mice (Gioia et al., 2023). Additionally, we observed a decrease in DCX+ immature neurons in the ventral hippocampus of VPA, PNE, and NLGN3-R451C KI mice, while no such change was observed in IQSEC2 KO mice. This indicates that the former models are more likely affected by impaired proliferation or differentiation of immature neurons, whereas the latter may involve disruption in apoptosis or survival of mature neurons. VPA causes syndromic effects and influences broad brain regions through its HDAC inhibitory activity, raising the possibility that the observed impairments in adult hippocampal neurogenesis in both the ventral and dorsal regions in our study may not solely reflect ASD-specific mechanisms but could result from generalized neurotoxic effects. However, our results suggest that disturbed adult neurogenesis in the ventral hippocampus may contribute to the neuronal mechanisms underlying social behavior deficits in ASD, despite the different underlying pathways.

One of the limitations of this research is the methodology to estimate the total numbers of the BrdU/NeuN double-positive cells and DCX positive cells in the brain. Recently, brain transparent and 3D-scanning techniques have been available to count all the cells in the whole brain (Mano et al., 2018). These methods will provide more information on the spatial distribution of adult neurogenesis in the hippocampus. Furthermore, considering the growing identification of genetic mutations in ASD patients, further research to include diverse genetic models will be crucial for deepening our understanding of ASD mechanisms.

## 5 Conclusion

In this study, we confirmed ASD-like social interaction deficit in mice treated with the prenatal exposure to valproic acid and nicotine. Additionally, we found that the adult neurogenesis in the ventral DG was impaired across several autistic mouse models, including IQSEC2 KO mice and NLGN3-R451C KI mice. These results suggest that disrupted adult neurogenesis in the ventral hippocampus may be a hallmark of ASD pathology.

## Generative AI statement

The authors declare that Gen AI was used in the creation of this manuscript. We used ChatGPT 4o to assist English grammatical corrections.

## Data Availability

The original contributions presented in the study are included in the article/supplementary material, further inquiries can be directed to the corresponding author.
